# An engineered GH1 β-glucosidase displays enhanced glucose tolerance and increased sugar release from lignocellulosic materials

**DOI:** 10.1038/s41598-019-41300-3

**Published:** 2019-03-20

**Authors:** Clelton A. Santos, Mariana A. B. Morais, Oliver M. Terrett, Jan J. Lyczakowski, Letícia M. Zanphorlin, Jaire A. Ferreira-Filho, Celisa C. C. Tonoli, Mario T. Murakami, Paul Dupree, Anete P. Souza

**Affiliations:** 1 Centro de Biologia Molecular e Engenharia Genética, Universidade Estadual de Campinas, Campinas, SP Brazil; 2 University of Cambridge, Department of Biochemistry, Cambridge, UK; 3 Laboratório Nacional de Ciência e Tecnologia do Bioetanol, Centro Nacional de Pesquisa em Energia e Materiais, Campinas, SP Brazil; 4 Natural Material Innovation Centre, University of Cambridge, Cambridge, UK; 5 OpenPlant Synthetic Biology Research Centre, Department of Plant Sciences, University of Cambridge, Cambridge, UK; 6 Laboratório Nacional de Biociências, Centro Nacional de Pesquisa em Energia e Materiais, Campinas, SP Brazil; 7 Departamento de Biologia Vegetal, Instituto de Biologia, Universidade Estadual de Campinas, Campinas, SP Brazil

## Abstract

β-glucosidases play a critical role among the enzymes in enzymatic cocktails designed for plant biomass deconstruction. By catalysing the breakdown of β-1, 4-glycosidic linkages, β-glucosidases produce free fermentable glucose and alleviate the inhibition of other cellulases by cellobiose during saccharification. Despite this benefit, most characterised fungal β-glucosidases show weak activity at high glucose concentrations, limiting enzymatic hydrolysis of plant biomass in industrial settings. In this study, structural analyses combined with site-directed mutagenesis efficiently improved the functional properties of a GH1 β-glucosidase highly expressed by *Trichoderma harzianum* (ThBgl) under biomass degradation conditions. The tailored enzyme displayed high glucose tolerance levels, confirming that glucose tolerance can be achieved by the substitution of two amino acids that act as gatekeepers, changing active-site accessibility and preventing product inhibition. Furthermore, the enhanced efficiency of the engineered enzyme in terms of the amount of glucose released and ethanol yield was confirmed by saccharification and simultaneous saccharification and fermentation experiments using a wide range of plant biomass feedstocks. Our results not only experimentally confirm the structural basis of glucose tolerance in GH1 β-glucosidases but also demonstrate a strategy to improve technologies for bioethanol production based on enzymatic hydrolysis.

## Introduction

The recalcitrance of lignocellulose materials is the major problem for the effective utilisation of woody feedstocks to produce biofuels and high-value plant-derived chemicals^1^. The remarkable recalcitrant nature of biomass has a strong impact on its enzymatic saccharification, preventing enzymes from accessing the lignocellulosic matrix^2,3^. Overcoming this physical barrier is the biggest challenge currently facing the development of second generation biofuels^4^. Although studies focusing on the engineering of plant cell walls for enhanced biofuel production^5–7^ have shed some light on the matter, the bioprospecting of microorganisms and the improvement and application of novel biocatalysts that can increase the efficiency of the saccharification of cellulosic substrates are still vast fields to be explored^1,8–10^.

Our laboratory has previously reported the crystal structure and biochemical characterisation of a glycoside hydrolase family 1 (GH1) β-glucosidase highly expressed by *Trichoderma harzianum* (ThBgl) under biomass degradation conditions^11^. β-glucosidase (EC 3.2.1.21) catalyses the hydrolysis of β-glucosidic linkages in aryl-, amino-, or alkyl-β-D-glucosides and di- or oligosaccharides at the nonreducing ends, releasing β-D-glucose monomers^12^. This class of enzymes has a critical role within the set of enzymes that compose the enzymatic cocktails designed for plant biomass hydrolysis, thereby alleviating the inhibition of other cellulases by cellobiose during the reaction^13,14^. Notwithstanding their biotechnological potential, β-glucosidases have shown a different degree of sensitivity to high glucose concentrations and may be inhibited by cello-oligosaccharides^15^. Improved β-glucosidase activity was one of the ways in which one of the most efficient commercial enzymatic cocktails (Cellic^®^ CTec3, Novozymes A/S, Denmark) available for bioethanol production, was recently boosted^16^.

Based on the effect of glucose on β-glucosidases activity, a classification attempt was recently proposed in which β-glucosidases were grouped as follows: glucose tolerant; inhibited by low concentrations of glucose; stimulated by low glucose concentrations and inhibited by high glucose concentrations; and not inhibited by high glucose concentrations^17^. ThBgl is easily inhibited by glucose^11^. During large-scale biomass conversion, high concentrations of glucose must be reached, which requires enzymes that maintain high catalytic rates under these conditions. The molecular basis of glucose tolerance in β-glucosidases is not completely known^17^; however, it has been correlated with structural features at the entrance channel to the active site rather than the active site itself^18^. A comparative structural analysis of GH1 and GH3 β-glucosidases revealed that glucose-tolerant GH1 β-glucosidases showed a deeper and narrower substrate channel than other β-glucosidases, and this channel restricts glucose access to the active site, which is inconsistent with the shallow pocket observed in GH3 β-glucosidases^18^.

In the present study, structural data combined with site-directed mutagenesis were employed to improve ThBgl properties. The ThBgl structure (PDB ID 5BWF) was used to engineer a high glucose-tolerant protein by substitution with two amino acid residues. The tailored ThBgl (ThBgl-Mut) crystal structure revealed that the selected mutations mimicked the active-site entrance of the highly glucose-tolerant GH1 β-glucosidase from *Humicola insolens* (HiBG), acting as gatekeepers, as postulated for the HiBG glucose tolerance mechanism^18^. In addition, the performance of ThBgl-WT and ThBgl-Mut in saccharification and simultaneous saccharification and fermentation using a wide range of plant biomass feedstocks was evaluated. Our structural and biochemical data revealed remarkable differences between the wild-type and tailored proteins, confirming the efficiency of the active-site redesign approach. Taken together, our findings experimentally demonstrate the importance of the active-site entrance for glucose tolerance in GH1 β-glucosidases and show a strategy to optimize the efficiency of an enzyme, making it more suitable for industrial applications, especially those related to cellulosic ethanol production.

## Methods

### Protein expression and purification

The gene encoding the wild-type GH1 β-glucosidase from *T. harzianum* (ThBgl-WT) was cloned into pET-28a(+) as described in a previous protocol^11^. The L167W/P172L ThBgl double mutant (ThBgl-Mut) was synthesized by Epoch Life Science, Inc. (Sugar Land, TX, USA) and subcloned into pET-28a(+). The recombinant plasmids for ThBgl-WT and ThBgl-Mut were transformed into the *Escherichia coli* Rosetta strain (Novagen, Darmstadt, Germany) for protein overexpression. The protein expression was achieved by culturing each transformed cell individually in 1L of LB broth supplemented with kanamycin (30 µg mL^−1^) and chloramphenicol (34 µg mL^−1^) at 37 °C and with shaking at 300 rpm. Once the bacterial cultures reached an OD_600_ of 0.8, the recombinant protein expression was induced by 0.4 mM IPTG, followed by cultivation for 18 h at 16 °C and 180 rpm. The induced cells were harvested by centrifugation (3000 *g*, 15 min, 4 °C), resuspended in 50 mL of buffer A (40 mM HEPES pH 7.5 and 150 mM NaCl) containing 1 mg mL^−1^ lysozyme and 1 mM PMSF (phenylmethanesulfonyl fluoride) and incubated for 30 min on ice. The lysed cells were disrupted by ultrasonication, and the soluble fraction was collected by centrifugation (27,000 *g*, 45 min, 4 °C). Protein purification was achieved using nickel affinity chromatography and gel filtration using a Ni Sepharose High Performance His-Trap column (GE Life Sciences) and a HiPrep 16/60 Sephacryl S-100 HR column (GE Life Sciences), respectively. All chromatographic steps were performed with columns coupled to an ÄKTA FPLC device (GE Life Sciences) or its equivalent, and the absorbance of eluted proteins was monitored at 280 nm. The concentrations of the purified proteins were determined using a Bradford protein assay (Sigma-Aldrich, UK), and the protein purity was estimated by SDS-PAGE.

### Biochemical studies

Initially, the optimal temperature and pH for ThBgl-WT and ThBgl-Mut proteins were evaluated using *p*-nitrophenyl-β-glucopyranoside (*p*NPG, Sigma-Aldrich; St. Louis, MO USA) as the substrate and 100 μL reactions containing 3 μg of purified enzyme, 100 mM buffer, pH 7.0, and 0.5 mM *p*NPG. All reactions were incubated for 10 min and stopped with the addition of 100 μL of 1M Na_2_CO_3_. The absorbance of the *p*-nitrophenol released during the reaction was measured at 405 nm using an Infinite^®^ 200 PRO microplate reader (TECAN, Männedorf, Switzerland). The optimal temperature was evaluated in assays ranging from 20 to 75 °C. The pH dependence of the enzymatic activity was determined in a pH range from 3.0 to 10.0 using the buffers citrate–phosphate (pH 3.0, 4.0, 4.5, 5.0, and 5.5), phosphate (pH 6.0, 6.5, 7.0, and 8.0), and glycine (pH 9.0 and 10.0) at a final concentration of 100 mM. Subsequent to the determination of the optimal temperature and pH, kinetic experiments were performed in 100 mM sodium phosphate buffer (pH 6.0) at the optimum temperature of each enzyme by monitoring the rate of hydrolysis of *p*NPG at concentrations ranging from 0.0 to 12.0 mM. The kinetic parameters (*K*_M_ and *V*_max_) were obtained using GraphPad Prism (GraphPad Software, San Diego, CA, USA) to nonlinearly fit the data with the Michaelis–Menten equation.

The inhibitory effect of glucose on both ThBgl-WT and ThBgl-Mut was investigated by measuring the enzymatic activity on the hydrolysis of *p*NPG at glucose concentrations ranging from 0.0 to 1.0M. The relative activity was defined as the enzymatic activity at different glucose concentrations relative to the enzymatic activity without glucose. All measurements were performed in triplicate and repeated at least twice.

### Crystallization, data collection and processing, and structure determination

ThBgl-Mut was concentrated to 11.5 mgmL^−1^ for crystallization trials. Crystals of ThBgl-Mut were grown by the sitting-drop vapor-diffusion method at 18 °C in drops containing 0.5 μL of the concentrated protein and the same volume of reservoir solution (20% (w/v) PEG 3350, 0.2M sodium nitrate, 0.1M Bis-tris propane pH 6.5).

X-ray diffraction data at a wavelength of 1.459 Å were collected at the W01B-MX2 beamline at the Brazilian Synchrotron Light Laboratory, Campinas, Brazil using a PILATUS2M detector (Dectris, Baden-Dattwil, CHE). Data processing was performed using the XDS package^19^. The ThBgl-Mut structure was solved by the molecular replacement method MOLREP^20^ using the wild-type enzyme ThBgl (PDB ID: 5BWF^11^) as a template. Refinement cycles were performed using PHENIX Refine^21^ alternated with manual inspection using the program COOT^22^. TLS groups used during refinement, were generated by the TLSMD server^23^.

The final model was validated using the program MolProbity^24^. Data collection, processing and refinement statistics are summarized in Table 1. Representations of the structures were generated in PyMOL^25^, and the structure superposition r.m.s.d. was calculated with PDBeFOLD^26^. The ThBgl-Mut structure was deposited in the RCSB Protein Data Bank (PDB) under the accession code 6EFU.

**Table 1:** Data collection and refinement statistics of ThBgl-Mut (Leu167Trp/Pro172Leu).

Data collection	
Space group	*P*2_1_2_1_2_1_
Cell dimensions	
a, b, c (Å)	91.61, 96.21, 96.45
Molecules per AU^a^	2
Resolution (Å)	48.22 – 2.2
Observed reflections	253,768 (25,032)
Unique reflections	43,877 (4,310)
CC_1/2_^b^	0.99 (0.45)
I/σI	9.74 (1.17)
Completeness (%)	99.87 (99.61)
R-meas	0.164
R-merge	0.149 (1.491)
Multiplicity	5.8 (5.8)
**Refinement**	
Resolution (Å)	48.22 – 2.2
No. reflections	43,852
R_work_/R_free_	0.178/0.221
No. protein residues	929
B-factor (Å^2^)	
Average	44.80
Macromolecules	44.90
Ligands	59.00
Solvent	38.80
Root mean square deviations	
Bond lengths (Å)	0.006
Bond angles (°)	0.79
Ramachandran Plot	
Favored (%)	96
Outliers (%)	0.11
MolProbity clashscore	2.79
PDB code	6EFU

Values in parentheses are for the highest-resolution shell. ^a^AU: asymmetric unit; ^b^CC_1/2_: correlation between intensities from random half-datasets^37^.

### Plant material and AIR preparation

Sugarcane (steam-exploded bagasse and pith), *Pinus sylvestris* (pine softwood) and birch wood chips (Sigma-Aldrich, UK) were used as feedstocks for the saccharification experiments. The sugarcane consisted of approximately 49.8% (w/v) cellulose, 22.1% (w/v) hemicellulose, and 23.5% (w/v) lignin^27^. Alcohol insoluble residue (AIR) preparation was performed using a ball-milling procedure and no additional chemical pretreatments as described previously^28^.

### Laboratory-scale enzymatic saccharification experiments

Saccharification reactions were performed using the combination of cellobiohydrolase I (CBHI) from *Trichoderma* sp. and endo-1, 4-β-D-glucanase (endocellulase) from *Aspergillus niger* (Megazyme, Wicklow, Ireland) supplemented with approximately 10 µg of ThBgl-WT or ThBgl-Mut proteins. For all plant biomass materials, enzymatic hydrolysis was carried out in sodium citrate buffer (100 mM, pH 6.0) in a final reaction volume of 0.5 mL using 0.5 mgmL^−1^ AIR material for 24 h at 45 °C at 1400 rpm of shaking applied for 30 s every 4 min using a ThermoMixer® C (Eppendorf, UK). The reaction was terminated by heat-treating the suspension at 100 °C for 10 min. Glucose release from the biomass samples was quantified using a commercial kit (K-GLUHK-220A; Megazyme, Wicklow, Ireland). The glucose concentration for each experiment was standardised with readings obtained using biomass and enzyme alone as controls. For each biological sample, at least 3 triplicates were analysed. Average sugar release for each assayed sample was used for the statistical analysis with Student’s t-test.

### Simultaneous saccharification and fermentation (SSF) experiments

For SSF experiments, 50 mg of ball-milled sugarcane bagasse without chemical pretreatments was used for each reaction and prepared as previously described by Lyczakowski *et al*.^6^. The fermentation reactions were amended with approximately 120 µg of ThBgl-WT or ThBgl-Mut in the presence of CBHI and endocellulase or only endocellulase, and 250 µL of a 0.6 OD_600_ TOP10 *E. coli* culture carrying the BBa_K1122676 BioBrick, which encodes a pyruvate decarboxylase and an alcohol dehydrogenase from *Zymomonas mobilis* that allow ethanol production in *E. coli*^29^. Control reactions using only biomass or biomass supplemented with CBHI and endocellulase or endocellulase alone were used to quantify the ethanol production in the presence of ThBgl enzymes. SSF reactions were carried out for 96 h at 37 °C and 200 rpm. Ethanol levels were analysed using a commercial kit (K-ETOH; Megazyme, Wicklow, Ireland). All reactions were performed in triplicate, and the average ethanol production for each assayed sample was used for the statistical analysis with Student’s t-test.

### Polysaccharide analysis by carbohydrate gel electrophoresis (PACE)

The products of the sugarcane steam-exploded bagasse saccharification reactions (0.5 mg) digested with ThBgl-WT or ThBgl-Mut in the presence of endocellulase enzyme were analysed by polysaccharide analysis using carbohydrate gel electrophoresis **(**PACE)^30^. Control hydrolysis reactions using endocellulase alone and no enzyme were also analysed. Released oligosaccharides were dried and reductively aminated with 8-aminonapthalene-1, 3, 6-trisulphonic acid (ANTS; Invitrogen) and separated by acrylamide gel electrophoresis using a Hoefer SE660 vertical slab gel electrophoresis apparatus (Amersham, Buckinghamshire, UK). Electrophoresis was performed at 10 °C using a 10% (w/v) polyacrylamide gel containing 0.5% (w/v) N,N-9-methylenebisacrylamide in a 0.1M Tris–borate pH 8.2 buffer system. PACE gels were run at 1000 V for 47 min and then visualized with a G:BOX (Syngene, Cambridge, UK) equipped with a short-pass detection filter (500–600 nm) and long-wave UV tubes (365 nm emission).

## Results

### Biochemical characterisation of ThBgl and its mutant reveal broad enzymatic differences

The double amino acid replacements L167W/P172L in the ThBgl-WT sequence were selected after comparative structural analyses (PDB ID 5BWF) with HiBG (PDB ID 4MDO), where these two amino acid residues were suggested to be gatekeepers involved in glucose tolerance^18^. The ThBgl double mutant L167W/P172L was overexpressed in and successfully purified from *E. coli* (Supplementary Fig. S1). Assays to find the optimum temperature and pH values were carried out using pNPG (Supplementary Fig. S2). A remarkable increase in the optimum temperature of ThBgl-Mut (50 °C) was observed when compared to ThBgl-WT (40 °C) (Supplementary Fig. S2a). Similarly, ThBgl-Mut showed an expanded range of pH-dependent activity and exhibited high activity from pH 4.0–9.0, which significantly differs from the pH range observed for ThBgl-WT (5.0–7.0) (Supplementary Fig. S2b).

Kinetic data revealed that ThBgl-Mut has a *K*_M_ of 0.41 ± 0.30 mM, which is less than one-half of the *K*_M_ of ThBgl-WT (1.17 ± 0.66 mM) (Supplementary Fig. S3). Moreover, the catalytic constant (*k*_cat_) was significantly higher in ThBgl-Mut (7.85 ± 0.22 s^−1^) than in ThBgl-WT (4.92 ± 0.54 s^−1^). In fact, the catalytic efficiency parameter (*k*_cat*/*_*K*_M_) of ThBgl-Mut is approximately 5-fold higher than that of ThBgl-WT under the conditions assayed.

**Figure 1:**
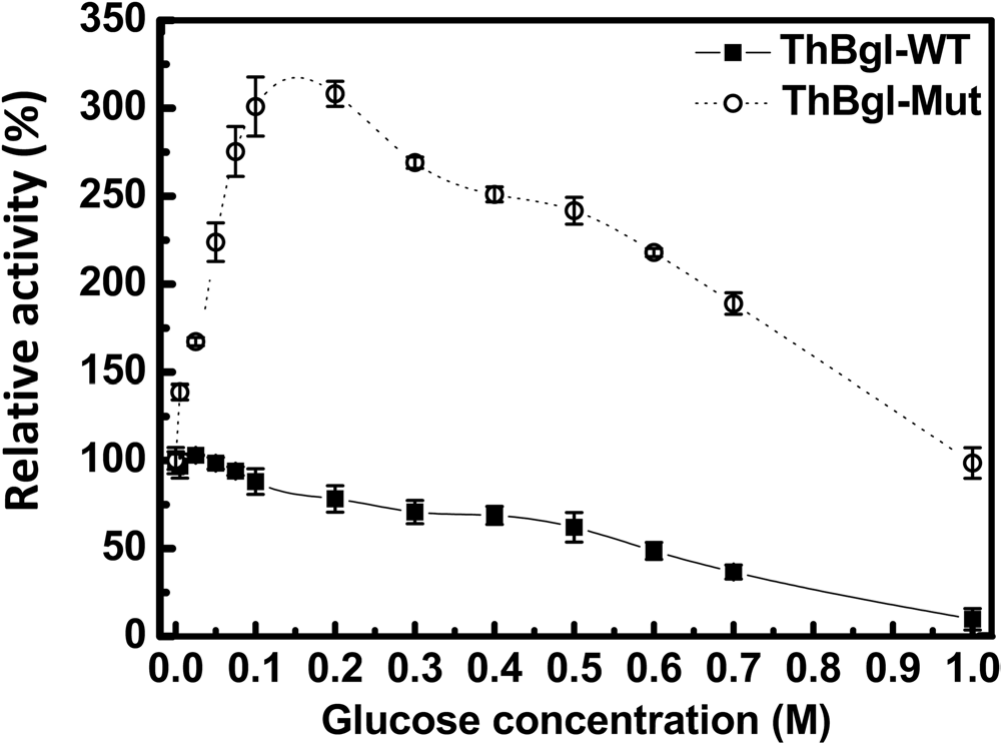
Effect of glucose on ThBgl-WT and ThBgl-Mut activities. Plots are the mean average of the relative activity of independent triplicates, and the error bars represent the standard deviations.

The inhibitory effect of glucose on the enzymatic activity of both ThBgl-WT and ThBgl-Mut was investigated at glucose concentrations up to 1.0M. The L167W/P172L double mutation had a significant impact on the glucose tolerance and stimulation of ThBgl (Fig. 1). In the presence of 0.1–0.25M glucose, although ThBgl-WT showed an accelerated decline in activity, ThBgl-Mut reached its highest levels, which represented an approximately 300% increase over the value measured under the no glucose conditions (Fig. 1). At the highest glucose concentration assayed (1.0M), the relative activity of ThBgl-Mut was still higher than that without the addition of glucose or than that at those concentrations where ThBgl-WT activity was stimulated (up to 0.05M glucose) (Fig. 1).

The kinetic and biochemical parameters observed for ThBgl-WT and its mutant corroborate those from a previous study in which a *Trichoderma reesei* β-glucosidase (TrBgl2) mutant L167W/P172L, which has 90% identity with ThBgl, was investigated^31^. However, great differences in the inhibitory effect of glucose were observed between ThBgl-Mut (Fig. 1) and the mutant TrBgl2 because ThBgl-Mut maintained high enzymatic activity at approximately 0.5M glucose, whereas a sharp decline in the activity of TrBgl2 at glucose concentrations higher than 0.1M was reported^31^. This finding demonstrates that ThBgl-Mut may be more suitable than other GH1 enzymes for biomass saccharification at high glucose concentrations.

### Engineered ThBgl-Mut mimics the active-site topology of GH1 glucose-tolerant β-glucosidases

**Figure 2:**
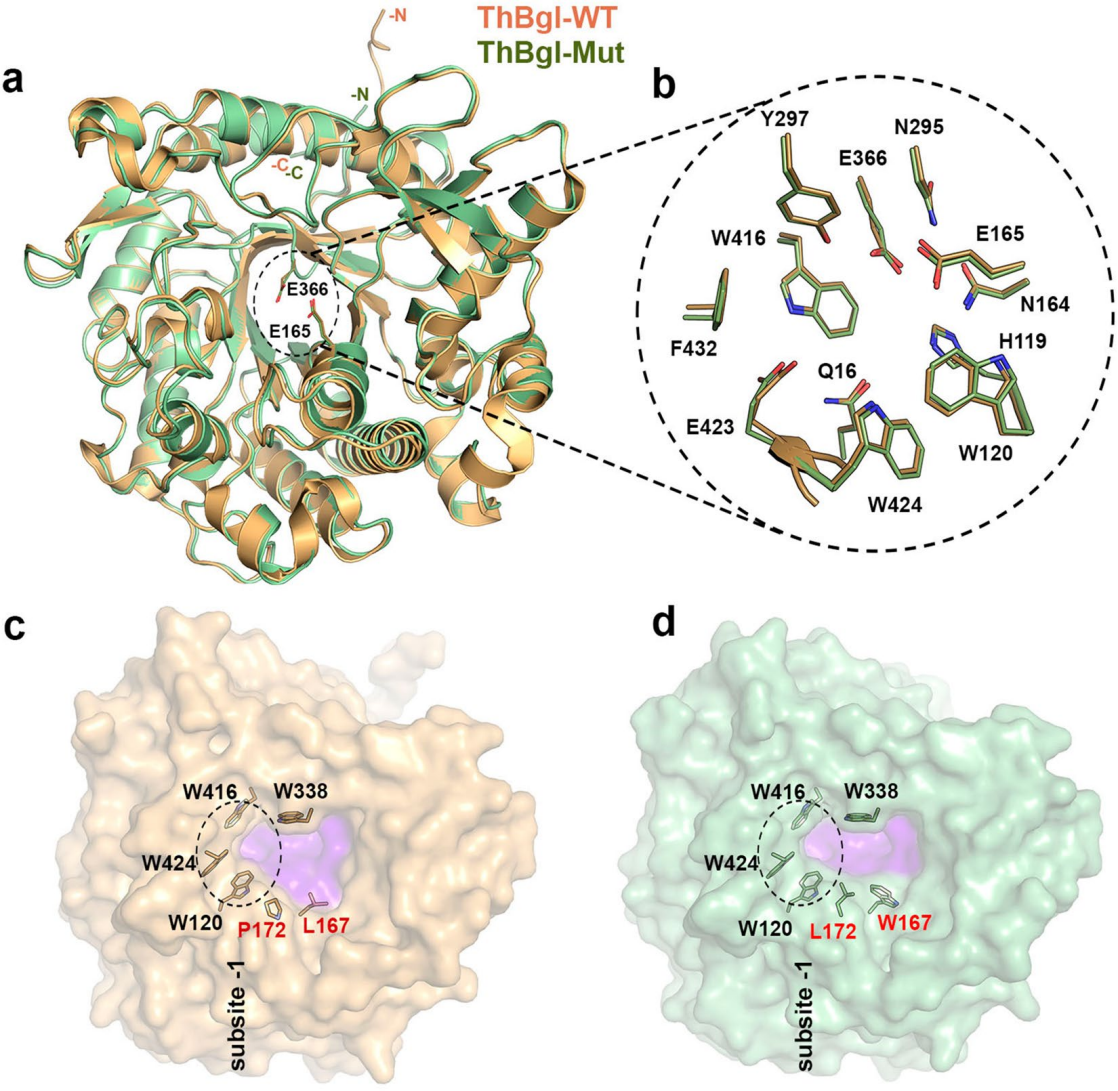
Structural comparison between ThBgl-WT and ThBgl-Mut. (**a**) Cartoon representation of ThBgl-WT (orange) and ThBgl-Mut (green), highlighting the predicted catalytic residues (sticks). N- and C-termini are indicated. (**b**) Enlarged view of amino acid residues from subsite -1, predicted to be important for catalysis^11^. (**c**) Surface representation of ThBgl-WT and ThBgl-Mut (**d**) highlighting the subsite -1 region represented in (**b**) with dashed lines and the active-site topology in light purple. The mutated residues (P172L and L167W) are labelled in red. Additionally, the positive-subsite delimiting residue W338 is represented as sticks.

A ThBgl-Mut orthorhombic crystal diffracted to 2.2 Å resolution, and two molecules were found in the asymmetric unit, as observed for ThBgl-WT^11^. As expected, the mutations did not affect the overall tertiary structure, thereby preserving the conserved GH1 (α/β)_8_-TIM barrel fold^32,33^. The ThBgl-Mut and ThBgl-WT structures are very similar (Fig. 2a) and display a r.m.s.d. value of 0.28 Å over 464 Cα atoms, with each monomer being considered. Moreover, the predicted catalytic residues E165 (acid–base) and E366 (nucleophile) are spatially conserved (Fig. 2a) and thus not affected by the rational modifications. Other putative catalytically important residues located in the glycone-binding site of ThBgl also have the same orientation in the WT and mutant enzymes (Fig. 2b).

**Figure 3:**
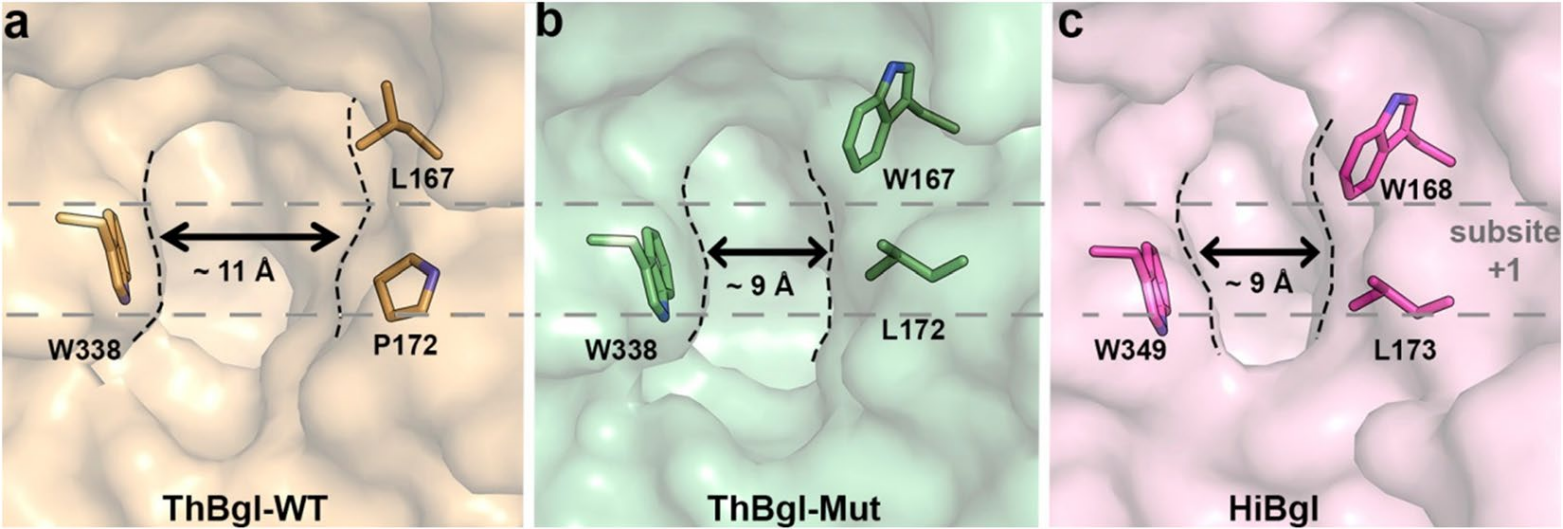
The engineered ThBgl-Mut active-site topology is similar to that of the glucose-tolerant HiBgl. (**a**) Surface representation of the ThBgl-WT (**a**) active-site topology, highlighting the conserved residue W338 and the mutated P172 and L167 residues. In ThBgl-Mut (**b**), these last two residues were replaced by L172 and W167, resulting in an active-site topology similar to that of HiBgl (**c**). The +1 subsite region is indicated by gray dashed lines. The estimated measures are an approximated average distance between the active-site extremities represented here.

As predicted by our structural analysis, the mutated residues W167 and L172 function as gatekeepers and affect the positive-subsite region topology, narrowing the ThBgl active-site pocket, while the −1 subsite is fully conserved (Figs 2c,d and 3). At the other extremities of these residues, the conserved W338 (corresponding to the residues W349 in HiBgl and W339 in TrBgl) delineates the new, narrowed entrance to the active site. The distance between the two sides of the active site entrance channel is clearly diminished by the ThBgl mutations, which explains its superb tolerance to high glucose concentrations (Fig. 3) and is fully in accordance with the proposed structural determinants for the glucose tolerance mechanism described for HiBgl^18^. Moreover, the same distances between the conserved Trp and the corresponding mutated residues were found in ThBgl-Mut and HiBgl active sites, explaining the similar glucose tolerance levels in these two enzymes (up to 450 mM in HiBgl and 500 mM in ThBgl-Mut) (Fig. 3).

### ThBgl-Mut increases glucose release from lignocellulosic materials

A wide range of plant biomass materials, including sugarcane, steam-exploded bagasse and raw material, together with pine and birch wood, were employed to compare the efficiency of ThBgl-WT and its mutant protein in laboratory-scale enzymatic saccharification experiments. All assays were performed using AIR without further chemical pretreatments under conditions of limited saccharification and no hemicellulases, such as xylanases, to reveal differences in the rate or extent of saccharification of cellulose with different enzyme cocktails. The β-glucosidases were used in conjunction with a CBHI and an endo-1, 4-β-D-glucanase, thus allowing the experiments to specifically focus on β-glucosidase activity.

**Figure 4:**
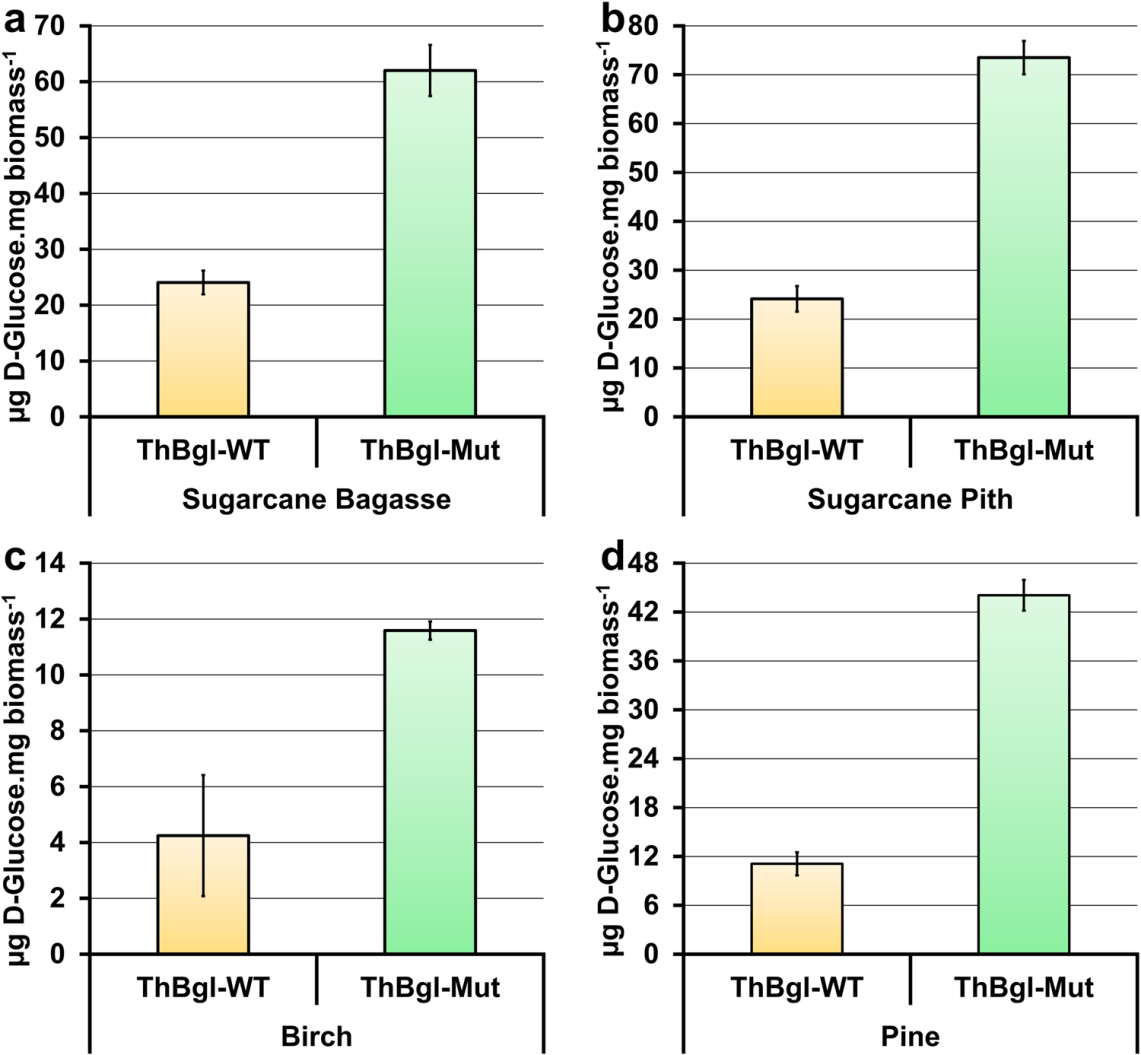
Comparative analysis of the performances of ThBgl-WT and ThBgl-Mut enzymes in laboratory-scale biomass saccharification assays. Saccharification assays were performed using ball-milled biomass material derived from (**a**) sugarcane steam-exploded bagasse, (**b**) sugarcane pith, (**c**) pine and (**d**) birch. Glucose release was quantified using a commercial kit. The detailed procedure is described in the ‘Methods’ section. Error bars indicate standard errors of the mean from triplicate experiments. All differences in sugar release between ThBgl-WT and ThBgl-Mut were statistically significant at a *p*-value ≤ 0.01.

A remarkable improvement in the glucose release from all lignocellulosic materials used was observed in saccharification reactions when ThBgl-Mut was used (Fig. 4). Initially, the time, temperature and protein loading for the enzymatic hydrolysis were optimized using sugarcane bagasse as a substrate (Supplementary Fig. S4). Despite a time dependence on the sugar release during the saccharification reactions, within 48 h, ThBgl-WT reached the same glucose release observed for ThBgl-Mut at 24 h (Supplementary Fig. S4a). This observation confirms the kinetic data, which show that ThBgl-Mut is a more efficient enzyme (Supplementary Fig. S3). ThBgl-WT showed no significant differences in glucose release at 40 °C and 45 °C and a reduced release at 50 °C, whereas ThBgl-Mut showed greater glucose release at 45 °C (Supplementary Fig. S4b). Concerning the dose effect of ThBgl-WT and ThBgl-Mut concentrations, varying the protein load from 10 to 100 µg did not produced significant increase in glucose release (Supplementary Fig. S4c). Therefore, all saccharification assays were performed at 45 °C for 24 h using 10 µg of ThBgl enzymes as established in the optimization experiments.

As expected, differences in the amount of glucose released varied with the sort of biomass tested (Fig. 4). For example, higher glucose yields were obtained for hydrolysis reactions using steam-exploded sugarcane bagasse and sugarcane pith compared with the use of other plant feedstocks. In the reactions using the sugarcane material the use of ThBgl-Mut, compared with ThBgl-WT, resulted in an approximately 300% increase in glucose release (Fig. 4a,b). The improvement in the ThBgl-Mut efficiency was also confirmed with the hardwood and softwood materials (Fig. 4c,d). Birch saccharification reactions released less glucose than other plant biomass materials; however, ThBgl-Mut still resulted in increased glucose release (Fig. 4c).

### ThBgl-Mut can boost bioethanol production

**Figure 5:**
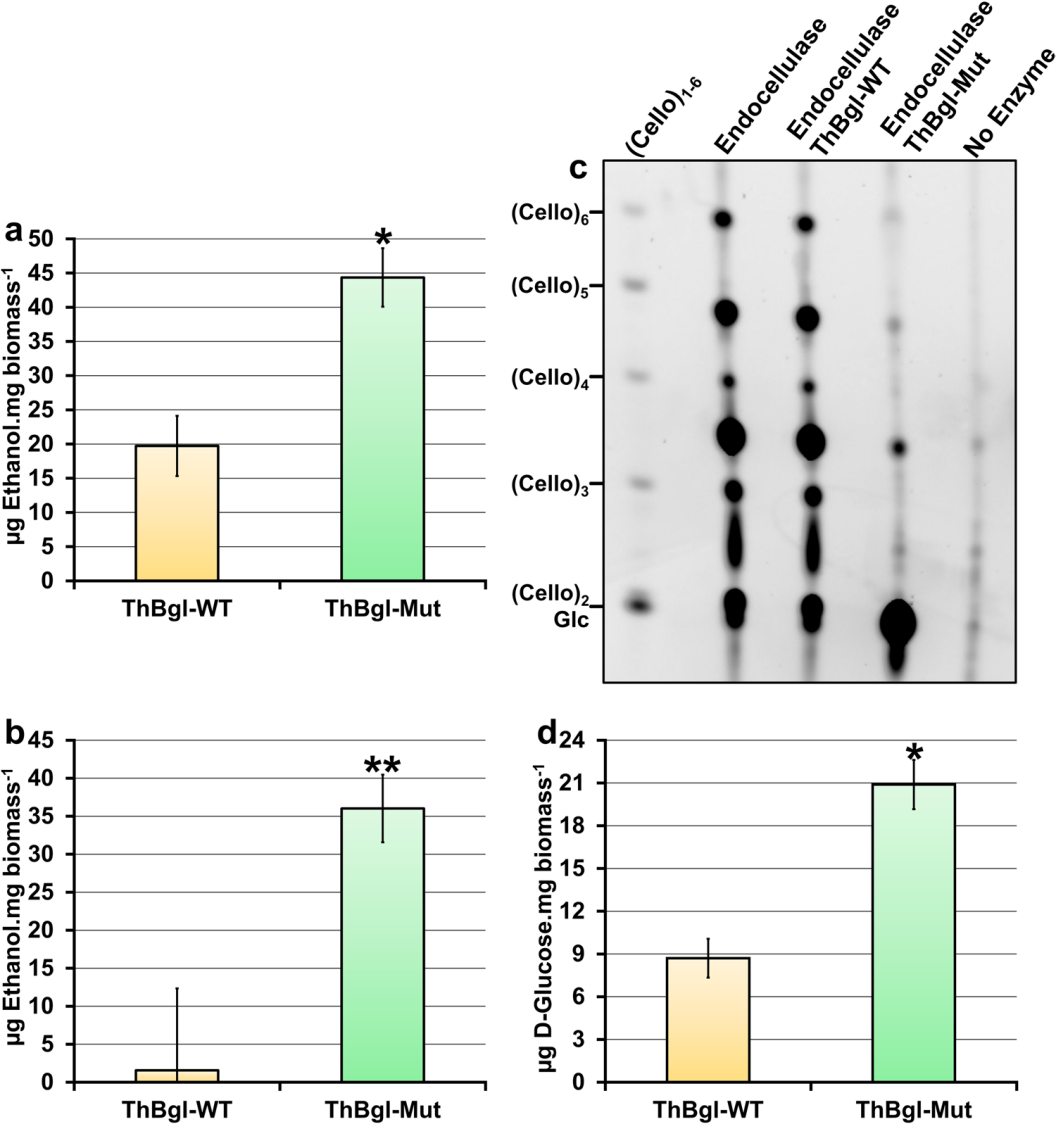
Bioethanol production and analysis of the cello-oligosaccharides generates by ThBgl-WT and ThBgl-Mut activities. The ethanol concentration after 96 h of SSF was measured (**a**) for ThBgl-WT or ThBgl-Mut in the presence of CBHI and endocellulase or (**b**) of only endocellulase. Ethanol yields were standardised using readings from a control fermentation reaction with only CBHI and endocellulase enzymes (Supplementary Table S1). (**c**) 10% PACE gel of sugarcane bagasse saccharification products generated by ThBgl-WT and ThBgl-Mut in the presence of endocellulase. Sugarcane bagasse digested using only endocellulase or in the absence of enzymes is also shown. A cello-oligosaccharide (Cello)_1 – 6_ ladder is shown on the left. Under these standard conditions, glucose (Glc) and cellobiose (Cello_2_) closely comigrate in the PACE. High-and low-contrast (exposure) gels are shown in Supplementary Fig. S7. (**d**) Glucose released from sugarcane bagasse saccharification performed using ThBgl-WT or ThBgl-Mut and endoglucanase. Error bars represent the standard deviation of triplicate experiments, *p-value ≤ 0.05; **p-value ≤ 0.01.

The influence of ThBgl-WT and ThBgl-Mut on bioethanol production was tested in SSF experiments using a synthetic bacterial system able to produce ethanol^29^. In a finding of special importance to biorefineries that use sugarcane bagasse for bioethanol production, our results showed that compared with ThBgl-WT, ThBgl-Mut boosted ethanol production two-fold (Fig. 5a). The initial PACE analysis of the cello-oligosaccharide products from sugarcane bagasse saccharification indicated notable differences between ThBgl-WT and ThBgl-Mut in the reactions supplemented only with endocellulase (Supplementary Fig. S5). SSF assays under these conditions were also performed. Our findings showed that ThBgl-Mut activity greatly differs from that of ThBgl-WT, enabling higher bioethanol yields, just as in the absence of CBHI (Fig. 5b).

To further clarify the increase in bioethanol production, especially in those SSF reactions exploring only the endo-1, 4-β-D-glucanase and β-glucosidase activities, the glucose release and the products of sugarcane bagasse saccharification under these conditions were analysed in detail via PACE^30^ (Fig. 5c). Our findings strongly support the hypothesis that ThBgl-Mut hydrolyses short cello-oligosaccharides more efficiently, thereby generating more glucose than the WT, as experimentally confirmed (Fig. 5d). Because of the observed difference between ThBgl-Mut substrate specificity and that of the WT enzyme, a possible synergistic action between ThBgl-WT and ThBgl-Mut to boost the saccharification yield was also investigated; however, no enhancement was observed (Supplementary Fig. S6). The ThBgl-Mut-dependent improvement of both sugar release and bioethanol yield seems to be coupled to the better utilization of biomass for enhancing enzyme activity and significantly differs from the behaviour of ThBgl-WT.

## Discussion

β-glucosidases that are highly tolerant to glucose inhibition and exhibit efficient hydrolytic properties are continually sought after for industrial applications^1,9^. Herein, we present new insights into glucose tolerance and demonstrate that the stimulation of GH1 β-glucosidase activity can be achieved by rationally converting a low glucose-tolerant enzyme into a high glucose-tolerant enzyme with improved catalytic activity on both pure carbohydrates and lignocellulosic materials. Furthermore, the differential enzymatic properties of ThBgl-WT and ThBgl-Mut in the laboratory-scale saccharification of a wide range of plant biomass materials and the impact of ThBgl-Mut on bioethanol production are presented for the first time.

Few studies have engineered fungal β-glucosidases to improve enzyme activity and thermostability; in addition, most of these studies were performed on *T. reesei* β-glucosidase^31,34^. Nevertheless, *T. reesei* shows weak β-glucosidase activity, and the use of its cellulases for biomass saccharification requires supplementation with additional β-glucosidases^34,35^.

Notwithstanding, consistent improvement of β-glucosidase activity has been achieved using site-directed mutagenesis based on high-resolution protein structures available in the PDB (www.rcsb.org). For example, after solving a protein structure, Lee and collaborators^34^ characterised a wide range of potential mutations in the substrate entrance of *T. reesei* β-glucosidase (TrBgl2; PDB ID 3AHY)^33^. Interestingly, the potential of L167W and P172L single mutations for improving the enzyme activity was highlighted; although L167W increased the optimum temperature by 10 °C and both constructs showed reduced *K*_M_ and the best enzymatic proprieties compared to wild-type TrBgl2, no glucose stimulation was observed; moreover, the double mutant L167W/P172L was not analysed^34^.

Subsequently, mutations in TrBgl2 were also generated by replacing the amino acid residues L167, P172 and P388^31^ with the corresponding residues W168, L173 and F348 in the HiBgl structure (PDB ID 4MDO)^18^. In this study, the L167W/P172L TrBgl2 double mutant was characterised^31^; however, the current study is the first to determine the crystal structure of both WT and mutant proteins and demonstrate how these mutations alter the active-site channel to mimic the entrance of high glucose-tolerant enzymes. Although ThBgl and TrBgl share high sequence identity and structural similarity, some residues surrounding the active-site are not conserved, such as P338 (replaced by F348 in HiBgl and S337 in ThBgl) (Supplementary Fig. S8). Because this residue is closely located to a conserved tryptophan that participates in the enclosure of the active site (W338 in ThBgl, W349 in HiBgl and W339 in TrBgl) and due to the influence of a proline to phenylalanine substitution in TrBgl catalysis, it is likely that these neighbouring residues also play a role in glucose tolerance, explaining the differences observed in glucose stimulation levels of TrBgl^31^ and ThBgl mutants.

In contrast to TrBgl-Mut, ThBgl-Mut showed continuous stimulation even at high glucose concentrations (1.0M), and the results were very similar to those observed in HiBgl^36^. The designed ThBgl mutations rendered its active-site topology very similar to that of HiBgl, in total accordance with the experimental observations regarding glucose tolerance and with the previously proposed glucose tolerance mechanism^18^. Parallel SSF experiments also demonstrated the improved performance of the ThBgl-Mut over the WT protein. Our findings show that the rational mutation approach used herein was able to enhance the glucose tolerance of ThBgl; however, the glucose tolerance itself does not seem to be the unique property that improves the ThBgl-Mut performance for biomass saccharification. ThBgl-Mut may more efficiently handle short cello-oligosaccharides and/or may remains active at high catalytic rates, thus reflecting the increased glucose release upon saccharification assays. Further experiments, including those in which the catalytic activity of enzymes against complex substrates can be evaluated at high concentrations of glucose, are still required to address these issues.

Together, our results not only experimentally demonstrate the mechanisms of glucose tolerance and stimulation in GH1 β-glucosidases but also shed light on the strategies for improving technologies based on enzymatic hydrolysis for bioethanol production.

## Supplementary information

Supplementary Info

## Data Availability

The coordinates and structure factors of ThBgl-Mut have been deposited in the RCSB Protein Data Bank under the accession code 6EFU.

## References

[1] Himmel ME (2007). Biomass recalcitrance: engineering plants and enzymes for biofuels production. Science.

[2] Chang VS, Holtzapple MT (2000). Fundamental factors affecting biomass enzymatic reactivity. Appl. Biochem. Biotechnol..

[3] Alvira P, Tomas-Pejo E, Ballesteros M, Negro MJ (2010). Pretreatment technologies for an efficient bioethanol production process based on enzymatic hydrolysis: A review. Bioresour. Technol..

[4] Hassan SS, Williams GA, Jaiswal AK (2018). Emerging technologies for the pretreatment of lignocellulosic biomass. Bioresour. Technol..

[5] Loque D, Scheller HV, Pauly M (2015). Engineering of plant cell walls for enhanced biofuel production. Curr. Opin. Plant. Biol..

[6] Lyczakowski JJ (2017). Removal of glucuronic acid from xylan is a strategy to improve the conversion of plant biomass to sugars for bioenergy. Biotechnol. Biofuels.

[7] Cai Y (2016). Enhancing digestibility and ethanol yield of *Populus* wood *via* expression of an engineered monolignol 4-O-methyltransferase. Nat. Commun..

[8] Morais, S. *et al*. Deconstruction of lignocellulose into soluble sugars by native and designer cellulosomes. *mBio* **3**, 10.1128/mBio.00508-12 (2012). 10.1128/mBio.00508-12 PMC3520109 23232718

[9] Adsul MG, Singhvi MS, Gaikaiwari SA, Gokhale DV (2011). Development of biocatalysts for production of commodity chemicals from lignocellulosic biomass. Bioresour. Technol..

[10] Horta MAC (2018). Network of proteins, enzymes and genes linked to biomass degradation shared by *Trichoderma* species. Sci. Rep..

[11] Santos CA (2016). Crystal structure and biochemical characterization of the recombinant ThBgl, a GH1 β-glucosidase overexpressed in *Trichoderma harzianum* under biomass degradation conditions. Biotechnol. Biofuels.

[12] Ketudat Cairns JR, Esen A (2010). beta-Glucosidases. Cell. Mol. Life Sci..

[13] Singhania RR, Patel AK, Sukumaran RK, Larroche C, Pandey A (2013). Role and significance of beta-glucosidases in the hydrolysis of cellulose for bioethanol production. Bioresour. Technol..

[14] Medve, J., Karlsson, J., Lee, D. & Tjerneld, F. Hydrolysis of microcrystalline cellulose by cellobiohydrolase I and endoglucanase II from *Trichoderma reesei*: adsorption, sugar production pattern, and synergism of the enzymes. *Biotech. Bioengine*. **59**, 621–634, https://doi.org/10.1002/(sici)1097-0290(19980905)59:5<621::Aid-bit13>3.0.Co;2-c (1998). 10099380

[15] Xiao Z, Zhang X, Gregg DJ, Saddler JN (2004). Effects of sugar inhibition on cellulases and beta-glucosidase during enzymatic hydrolysis of softwood substrates. Appl. Biochem. Biotechnol..

[16] Cellulosic Ethanol Novozymes Cellic® CTec3 - secure your plant’s lowest total cost. *Novozymes A/S · Luna 2012-01394-01* **Application Sheet**, 6. http://s3.amazonaws.com/zanran_storage/bioenergy.novozymes.com/ContentPages/2546502386.pdf.

[17] Salgado JCS, Meleiro LP, Carli S, Ward RJ (2018). Glucose tolerant and glucose stimulated beta-glucosidases - a review. Bioresour. Technol..

[18] de Giuseppe PO (2014). Structural basis for glucose tolerance in GH1 β-glucosidases. Acta. Crystallogr. D. Biol. Crystallogr..

[19] Kabsch W (2010). XDS. Acta. Crystallogr. D. Biol. Crystallogr..

[20] Vagin A, Teplyakov A (1997). MOLREP: an Automated Program for Molecular Replacement. J. Appl. Crystallogr..

[21] Afonine PV (2012). Towards automated crystallographic structure refinement with phenix.refine. Acta. Crystallogr. D. Biol. Crystallogr..

[22] Emsley P, Cowtan K (2004). Coot: model-building tools for molecular graphics. Acta. Crystallogr. D. Biol. Crystallogr..

[23] Painter J, Merritt EA (2006). TLSMDweb server for the generation of multi-group TLS models. J. Appl. Crystallogr..

[24] Chen VB (2010). MolProbity: all-atom structure validation for macromolecular crystallography. Acta. Crystallogr. D. Biol. Crystallogr..

[25] DeLano, W. L. The PyMOL molecular graphics system v. 1.3r1 (Schrodinger, LLC, New York, 2010).

[26] Krissinel E, Henrick K (2004). Secondary-structure matching (SSM), a new tool for fast protein structure alignment in three dimensions. Acta. Crystallogr. D. Biol. Crystallogr..

[27] Camassola M, Dillon AJP (2012). Steam-exploded sugar cane bagasse for on-site production of cellulases and xylanases by *Penicillium echinulatum*. Energy & Fuels.

[28] Mortimer JC (2010). Absence of branches from xylan in Arabidopsis *gux* mutants reveals potential for simplification of lignocellulosic biomass. Proc. Natl. Acad. Sci. USA.

[29] Lewicka AJ (2014). Fusion of pyruvate decarboxylase and alcohol dehydrogenase increases ethanol production in *Escherichia coli*. ACS Synth Biol.

[30] Goubet F, Jackson P, Deery MJ, Dupree P (2002). Polysaccharide analysis using carbohydrate gel electrophoresis: a method to study plant cell wall polysaccharides and polysaccharide hydrolases. Anal. Biochem..

[31] Guo B, Amano Y, Nozaki K (2016). Improvements in Glucose Sensitivity and Stability of Trichoderma reesei beta-Glucosidase Using Site-Directed Mutagenesis. PLoS One.

[32] Henrissat B (1995). Conserved catalytic machinery and the prediction of a common fold for several families of glycosyl hydrolases. Proc. Natl. Acad. Sci. USA.

[33] Jeng WY (2011). Structural and functional analysis of three beta-glucosidases from bacterium Clostridium cellulovorans, fungus Trichoderma reesei and termite Neotermes koshunensis. J. Struct. Biol..

[34] Lee HL, Chang CK, Jeng WY, Wang AH, Liang PH (2012). Mutations in the substrate entrance region of beta-glucosidase from Trichoderma reesei improve enzyme activity and thermostability. Protein Eng. Des. Sel..

[35] Takashima S, Nakamura A, Hidaka M, Masaki H, Uozumi T (1999). Molecular cloning and expression of the novel fungal beta-glucosidase genes from Humicola grisea and Trichoderma reesei. J. Biochem..

[36] Souza FHM (2010). Purification and biochemical characterization of a mycelial glucose- and xylose-stimulated β-glucosidase from the thermophilic fungus *Humicola insolens*. Process Biochem..

[37] Karplus PA, Diederichs K (2012). Linking crystallographic model and data quality. Science.

