# Supplementary material for: An engineered GH1 β-glucosidase displays enhanced glucose tolerance and increased sugar release from lignocellulosic materials
Source: Sci Rep. 2019 Mar 20;9:4903. doi: 10.1038/s41598-019-41300-3 (PMC6426972; doi:10.1038/s41598-019-41300-3)
Supplement: Supplementary file 1 — Supplementary Info [file 41598_2019_41300_MOESM1_ESM.pdf]

An engineered GH1 β-glucosidase displays enhanced glucose tolerance and increased sugar release from lignocellulosic materials

Clelton A. Santos[1,2], Mariana A. B. Morais[3], Oliver M. Terrett[2], Jan J. Lyczakowski[2,4,5], Letícia M. Zanphorlin[3], Jaire A. Ferreira-Filho[1], Celisa C. C. Tonoli[6], Mario T. Murakami[3], Paul Dupree[2,4,5], Anete P. Souza[1,7*]

[1]*Centro de Biologia Molecular e Engenharia Genética, Universidade Estadual de Campinas, Campinas, SP, Brazil*

[2]*University of Cambridge, Department of Biochemistry, Cambridge, UK*

[3]*Laboratório Nacional de Ciência e Tecnologia do Bioetanol, Centro Nacional de Pesquisa em Energia e Materiais, Campinas, SP, Brazil*

[4]*Natural Material Innovation Centre, University of Cambridge, Cambridge, UK*

[5]*OpenPlant Synthetic Biology Research Centre, Department of Plant Sciences, University of Cambridge, Cambridge, UK*

[6]*Laboratório Nacional de Biociências, Centro Nacional de Pesquisa em Energia e Materiais, Campinas, Campinas, SP, Brazil*

[7]*Departamento de Biologia Vegetal, Instituto de Biologia, Universidade Estadual de Campinas, Campinas, SP, Brazil*

*Corresponding author

SUPPLEMENTARY INFORMATION

Supplementary Table S1;

Supplementary Figures S1-S8.

Sample	Average ethanol production ($\mu g.mg^{-1}$ biomass, s.d.*)
Control 1 – Cells	58.82 ± 12.12
Control 2 – CBH1 and GH45	111.76 ± 20.28
ThBgl-WT, CBH1 and GH45	131.50 ± 4.40
ThBgl-Mut, CBH1 and GH45	156.12 ± 4.28
Control 1 – Cells	52.12 ± 0.89
Control 2 – GH45	53.69 ± 14.01
ThBgl-WT, GH45	55.25 ± 10.77
ThBgl-Mut, GH45	89.71 ± 4.45

Supplementary Table S1. Average ethanol production during sugarcane bagasse simultaneous saccharification and fermentation assays. *s.d, standard deviation.

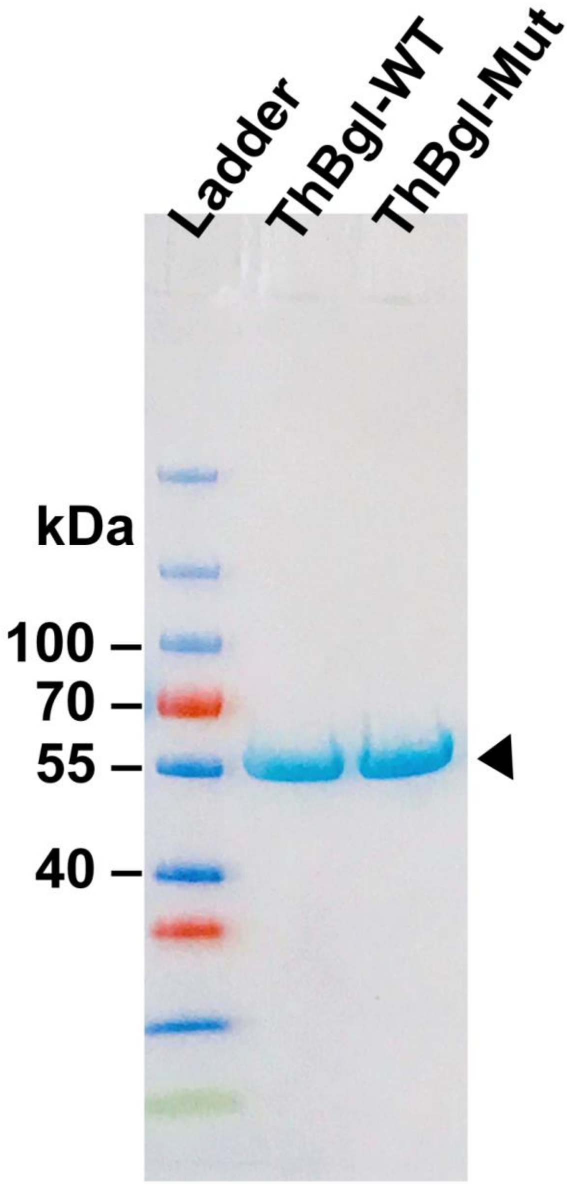

Supplementary Fig. S1. SDS-PAGE analysis of purified ThBgl-WT and ThBgl-Mut proteins. Approximately 5 µg of each protein was loaded on the gel. A protein molecular weight ladder is indicated on the left.

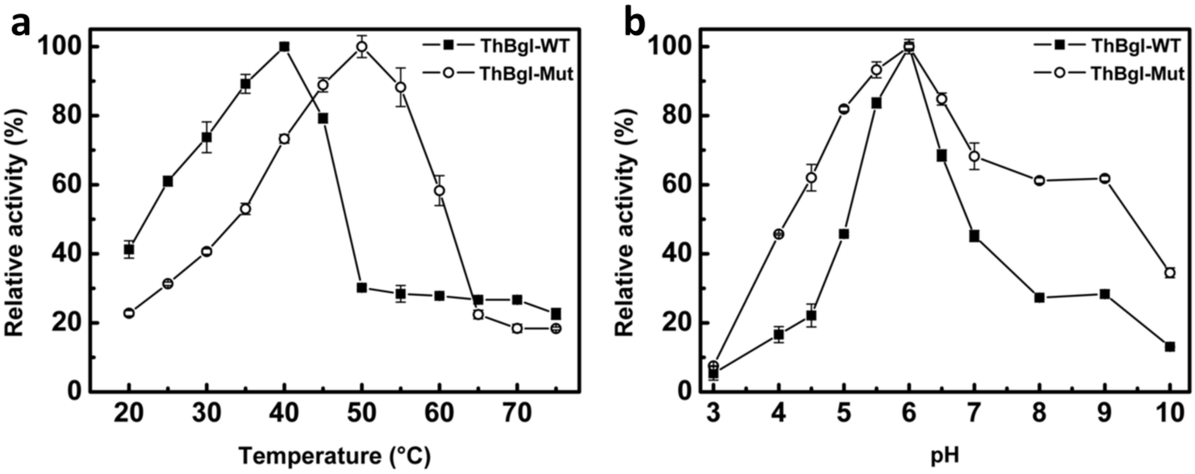

Supplementary Fig. S2. Biochemical properties of ThBgl-WT and ThBgl-Mut using *p*NPG as a substrate. (a) Optimal temperature screening (b) pH dependence analysis in a 100 mM citrate-phosphate-glycine buffer with a pH range from 3 to 10.

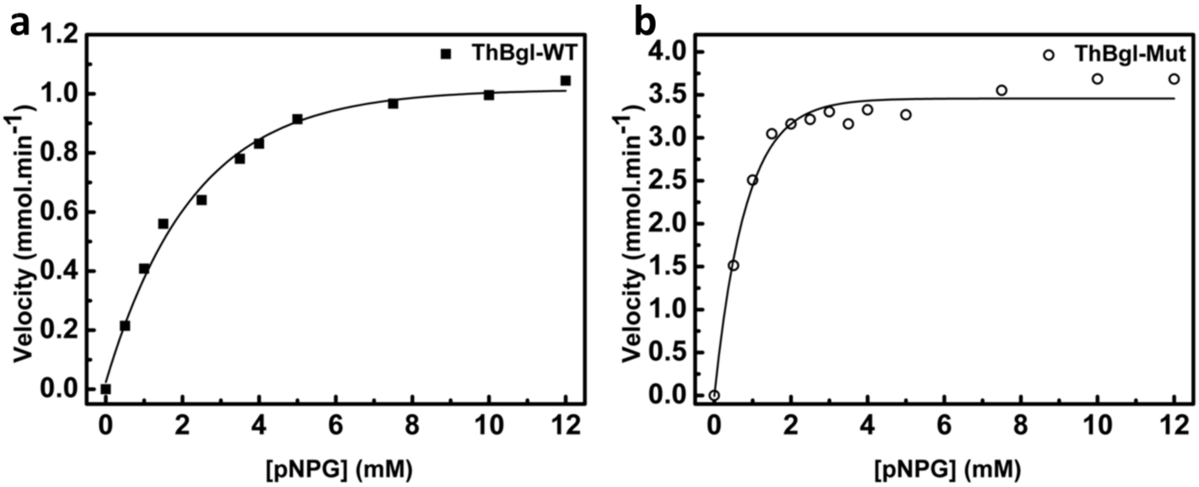

Supplementary Fig. S3. Kinetic properties of ThBgl-WT and ThBgl-Mut. (a) ThBgl-WT and (b) ThBgl-Mut kinetic parameters were obtained using a reaction mixture containing 100 mM sodium phosphate buffer with a pH of 6 and 10 mM *p*NPG as a substrate.

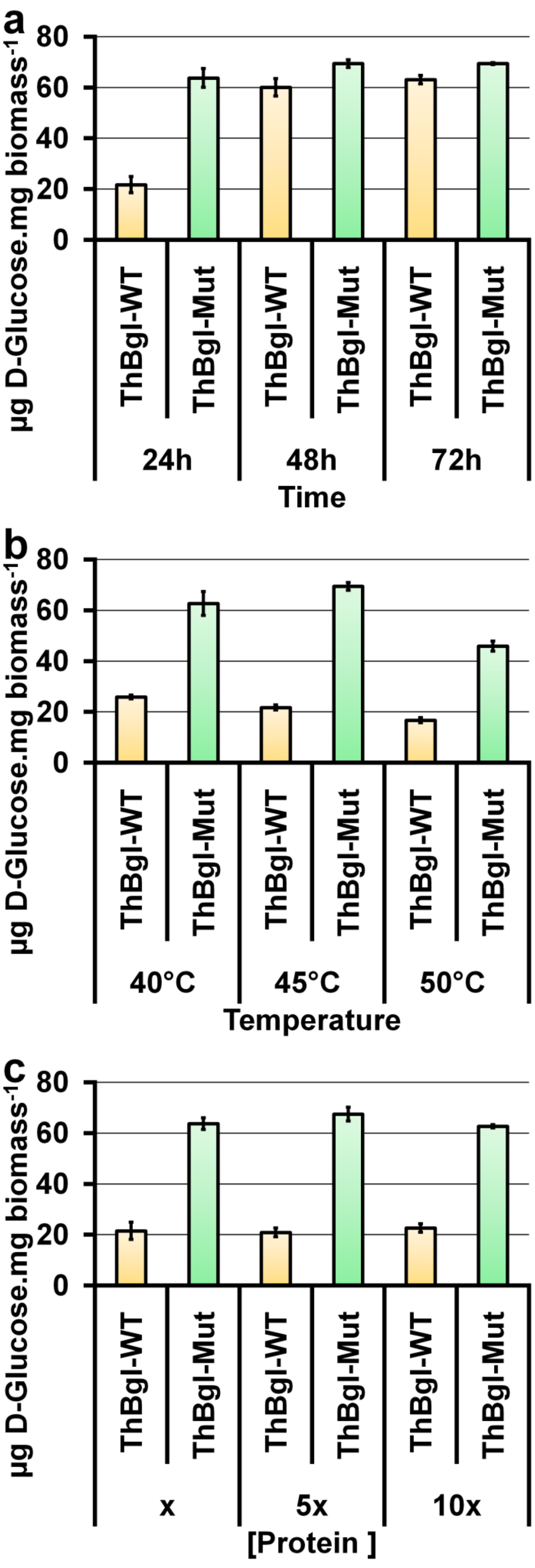
a
b
c
µg D-Glucose.mg biomass-1
80
60
40
20
0
ThBgl-WT
ThBgl-Mut
24h
48h
72h
Time
40°C
45°C
50°C
Temperature
x
5x
10x
[Protein]

Supplementary Fig. S4. Sugarcane bagasse saccharification optimisation experiments. Saccharification assays were performed using raw ball-milled sugarcane bagasse, and the effect of (a) time, (b) temperature and (c) enzyme loadings on the release of glucose was analysed. Glucose release was quantified using a commercial kit. The detailed procedure is described in the 'Methods' section. Error bars indicate standard errors of the mean from triplicate experiments.

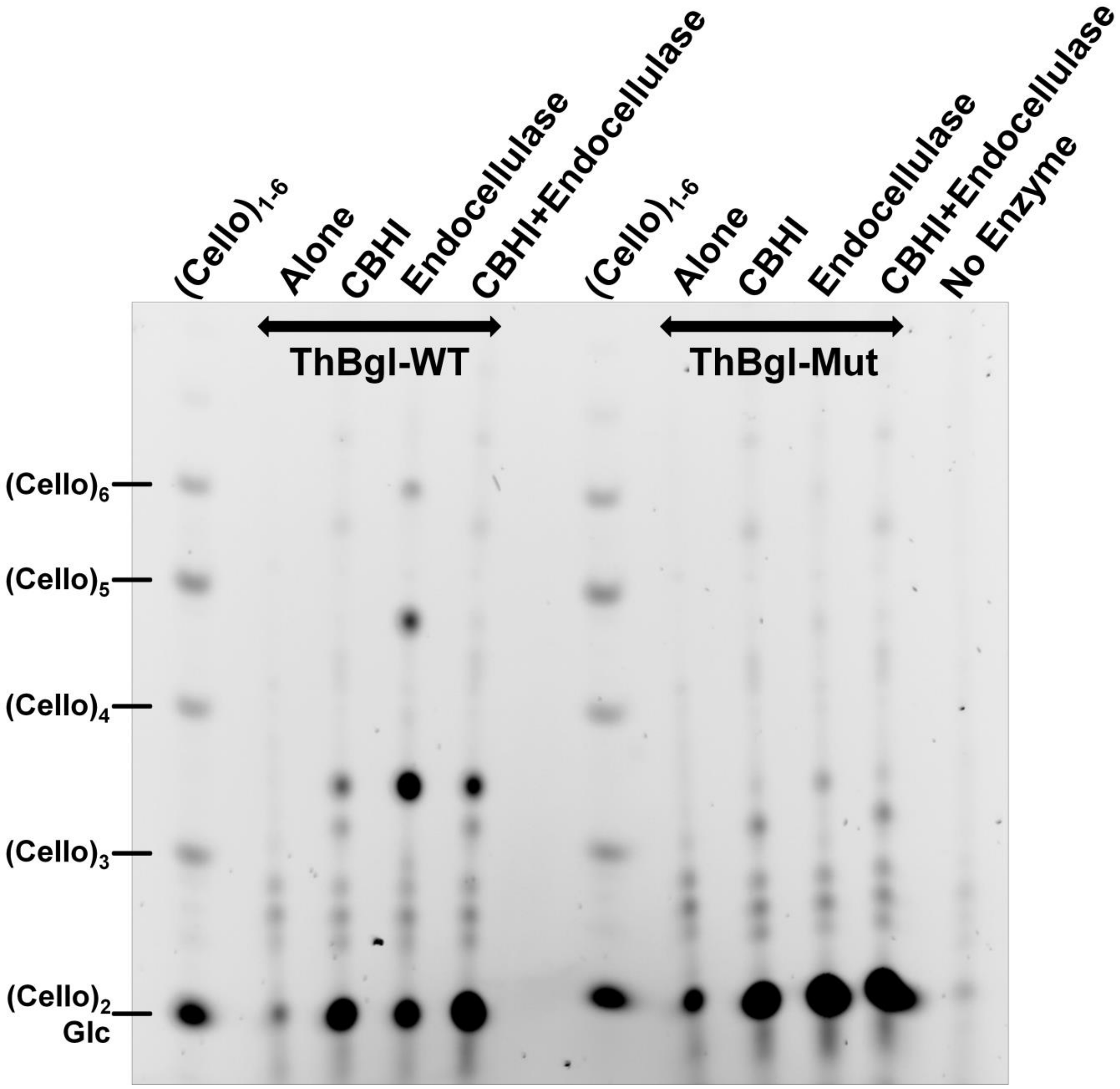

Supplementary Fig. S5. PACE gel analysis of cello-oligosaccharides after saccharification reaction using ThBgl-WT and ThBgl-Mut. The products of sugarcane bagasse digested using only CBHI or endo-cellulase or both enzymes were dried, reductively aminated with ANTS and separated by 10% PACE gel. Controls in the absence of enzymes are also shown. A cello-oligosaccharide $(Cello)_{1-6}$ ladder is shown on the left and in the middle of gel.

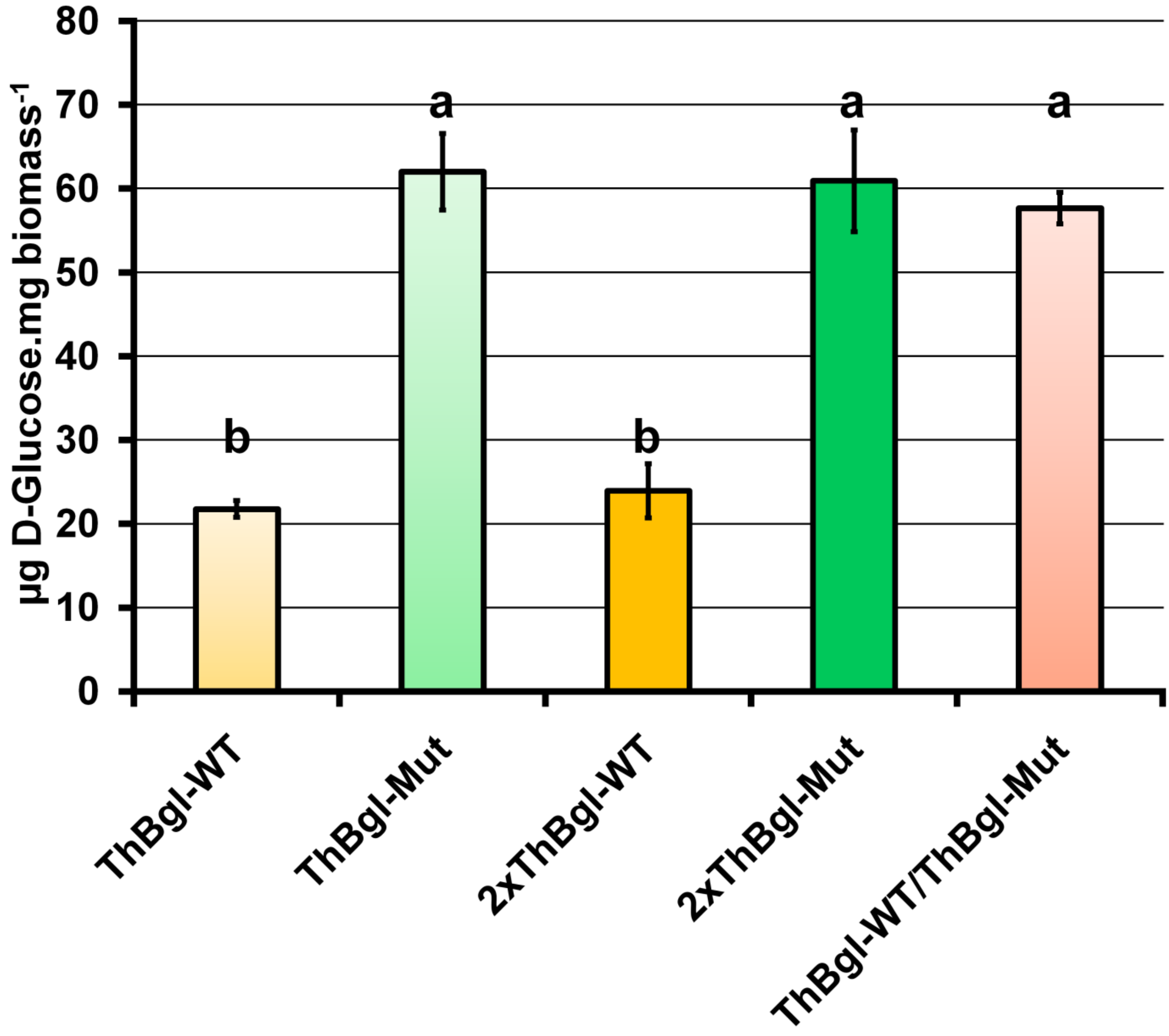

Supplementary Fig. S6. Synergistic action of ThBgl-WT and ThBgl-Mut in sugarcane bagasse saccharification experiments. The glucose release by ThBgl-WT or ThBgl-Mut alone in the presence of CBHI and endo-cellulase was compared with that of experiments in which both ThBgl enzymes were added in the same reaction. Error bars indicate standard errors of the mean from triplicate experiments. Different letters indicate significant differences in mean values (*p*-value ≤0.05).

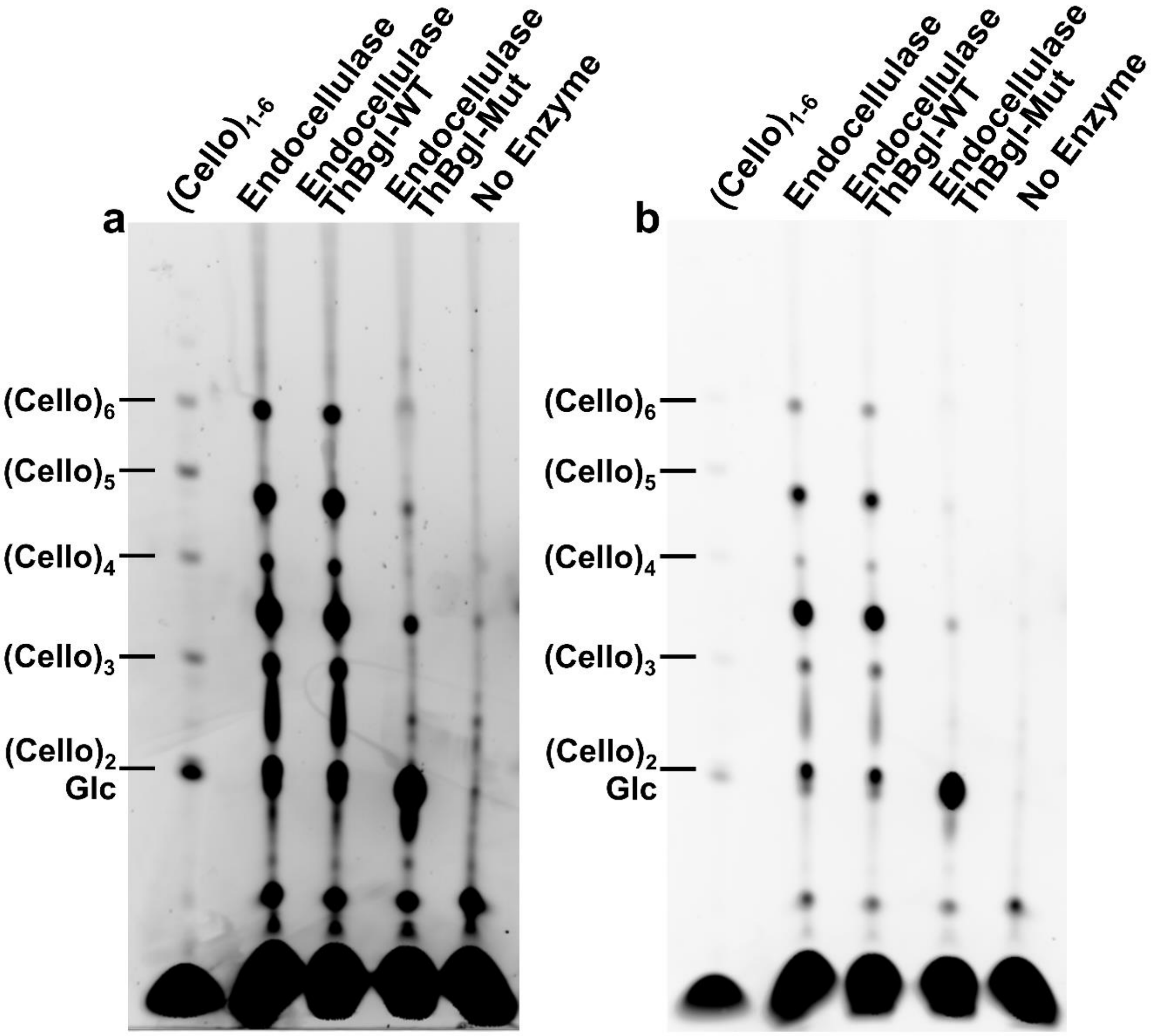

Supplementary Fig. S7. High- and low-contrast PACE gel analysis for the Figure 5c. (a) PACE gel visualised at 5 s exposure. (b) PACE gel visualised at 500 ms exposure. ANTS reaction excess is visualized in the basal part of the gel.

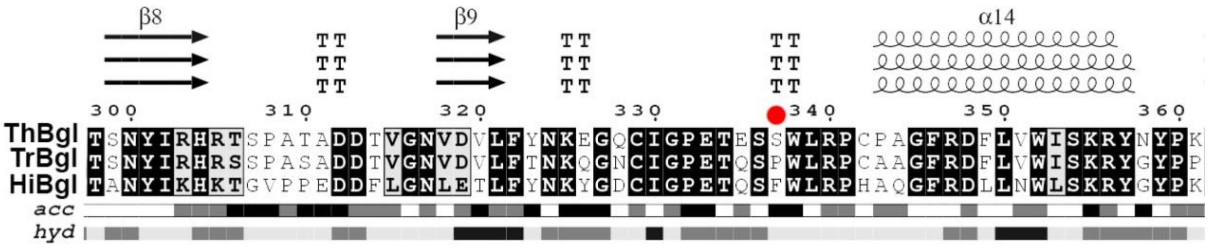

Supplementary Fig. S8. Amino acid sequence alignment of ThBgl (PDB ID: 5BWF), TrBgl (PDB ID: 3AHY) and HiBgl (PDB ID: 4MDO). The red sphere indicates the S337 of ThBgl, which is replaced by a Pro in TrBgl and by a Phe in HiBgl, followed by the conserved Trp. Acc = solvent accessibility colored from white (buried) to black (exposed). Secondary structure elements are shown above the alignment and labeled according to the ThBgl structure.
